# Implications of a Family History of Diabetes and Rapid eGFR Decline in Patients With Type 2 Diabetes and Biopsy-Proven Diabetic Kidney Disease

**DOI:** 10.3389/fendo.2019.00855

**Published:** 2019-12-13

**Authors:** Yiting Wang, Lijun Zhao, Junlin Zhang, Yucheng Wu, Rui Zhang, Hanyu Li, Ruikun Guo, Qianqian Han, Tingli Wang, Lin Li, Shanshan Wang, Fang Liu

**Affiliations:** ^1^Division of Nephrology, West China Hospital of Sichuan University, Chengdu, China; ^2^Division of Pathology, West China Hospital of Sichuan University, Chengdu, China

**Keywords:** diabetes, diabetic kidney disease, family history, end stage kidney disease, risk factors

## Abstract

**Objective:** This study aimed to identify the risk factors for a rapid decline in the estimated glomerular filtration rate (eGFR) in Chinese patients with type 2 diabetes and biopsy-proven diabetic kidney disease (DKD).

**Method:** This was a retrospective cohort study. Patients with biopsy-proven DKD who had been followed-up for at least 1 year were enrolled. Baseline clinicopathological data and serum creatinine levels that had been measured at least three times during follow-up in our hospital were collected. Patients were allocated to two groups of rapid decliners and slow decliners according to the median eGFR slope. The associations between potential risk factors and rapid eGFR decline were analyzed using logistic regression.

**Results:** A total of 128 eligible patients were enrolled and they had a mean age of 51.5 ± 10.7 years. During a median follow-up of 2 years, the median eGFR slope was −8.1 ± 14.4 mL/min/1.73 m^2^/year. The eGFR decline was significantly faster in patients with a family history of diabetes in first-degree relatives, nephrotic-range proteinuria, higher grades of glomerular pathology, and interstitial inflammation. No differences in the rate of the eGFR decline were observed in subgroups created according to sex, age, hypertension, glycosylated hemoglobin, diabetic retinopathy, interstitial fibrosis, and tubular atrophy. Logistic regression indicated that a family history of diabetes was independently associated with a rapid decline in eGFR, even after adjustment for factors including baseline eGFR and proteinuria.

**Conclusion:** A family history of diabetes in first-degree relatives is independently associated with a rapid decline in eGFR in the current relatively young studied patients. Our findings suggested that early diagnosis and treatment is important for these patients.

## Introduction

Diabetic kidney disease (DKD) is a common microvascular complication of diabetes. Management of DKD and the use of renin-angiotensin-aldosterone system (RAAS) inhibitors is beneficial for treating hyperglycemia and hypertension. However, DKD remains the leading cause of end-stage kidney disease (ESKD) worldwide (1–3). A decline in the glomerular filtration rate (GFR) and albuminuria are the predominant clinical features of DKD (4, 5). However, with extensive use of RAAS inhibitors, determining the decline in the estimated GFR (eGFR) appears to be a more robust method of predicting kidney dysfunction than albuminuria measurement. Previous studies have highlighted the association between the heterogeneity of a decline in eGFR trajectories and the risk of ESKD or mortality in patients with type 1 or type 2 diabetes (6–8). The goal of protecting kidney function in the early stage of DKD has motivated a search for risk factors affecting its susceptibility, initiation, and progression. The genetic and ethnic background, duration of diabetes, glycemia and blood pressure control, and albuminuria are associated with a decline in eGFR in patients with DKD (5, 8–11). However, most participants in previous studies had diabetes and kidney diseases, and inclusion of patients with non-diabetic kidney diseases may have confounded the findings.

The sequential pathological changes that occur in DKD are thought to be glomerular basement membrane thickening, mesangial expansion, nodular sclerosis (Kimmelstiel-Wilson lesion), and global glomerulosclerosis. Nodular glomerulosclerosis and global glomerulosclerosis are the major pathological lesions characterizing advanced DKD (12, 13). However, to better understand the course of DKD, whether the degrees of severity of these pathological lesions is associated with kidney function needs to be determined. To date, limited studies have evaluated the association between the pathological lesions of DKD and the rate of a decline in eGFR. Recently, a study of 37 Chinese ethnic patients with type 2 diabetes and biopsy-proven DKD showed that patients with glomerulosclerosis presented with a more rapid decline in eGFR (10). However, larger biopsy-proven DKD cohorts are required to investigate this finding in greater depth.

We conducted a retrospective cohort study to determine the trajectories of eGFR decline in patients with type 2 diabetes and biopsy-proven DKD. We also aimed to identify the clinical and pathological risk factors associated with a rapid decline in eGFR.

## Method

### Study Population

We performed a retrospective cohort study. The study included patients with type 2 diabetes and DKD who had undergone kidney biopsy in West China Hospital between 2007 and 2017. The diagnostic criteria for type 2 diabetes were based on the recommendations of the American Diabetes Association (14). Information regarding a family history of diabetes in first-degree relatives of the patients was collected. To accurately characterize trajectories of eGFR decline, we restricted our analysis to the following patients: (1) those in whom serum creatinine levels had been measured at least three times during the follow-up period; (2) all serum creatinine assays had been performed in our hospital to minimize methodological differences; and (3) the follow-up period was for at least 1 year (the follow-up period was calculated as the time from kidney biopsy until the last available serum creatinine measurement or an eGFR≤15 mL/min/1.73 m^2^). Patients who had progressed to ESKD at baseline were excluded from the study.

The study protocol conformed with the Declaration of Helsinki and was approved by the Ethics Committee of West China Hospital.

### Clinical and Renal Pathological Variables

Clinical data were collected from the electronic records system of our hospital. The eGFR was calculated using the Chronic Kidney Disease Epidemiology Collaboration creatinine formula. The trajectories of eGFR decline were evaluated using a simple linear model. Patients were defined as relatively rapid eGFR decliners (*n* = 64) and relatively slow eGFR decliners (*n* = 64), according to the median eGFR slope value (−8.1 mL/min/1.73 m^2^/year). We did not define “rapid eGFR decliners” as patients who had an eGFR slope < −5 mL/min/1.73 m^2^/year because more than 70% of the patients in our hospital display such a decline. Kidney biopsy specimens were routinely processed for light microscopy, immunofluorescence, and electron microscopy, and the pathological lesions were graded by at least two pathologists. The pathological classifications of glomerular alterations, interstitial fibrosis and tubular atrophy (IFTA), interstitial inflammation, and arteriolar hyalinosis were based on the criteria published by the Renal Pathology Society (12).

### Statistical Analysis

Data are shown as mean ± standard deviation or median and interquartile range, as appropriate. Differences between groups were analyzed using the Student's *t*-test, the Mann–Whitney test, one-way ANOVA or the chi-square test as appropriate. Univariable and multivariable logistic regression was used to evaluate the association between risk factors and a rapid decline in eGFR. All analyses were conducted using SPSS software 22.0 (IBM Inc., Armonk, NY, USA) and GraphPad Prism 7.0. A two-sided *P* < 0.05 was considered to represent statistically significance.

## Results

### Distribution of the Annual Decline in eGFR

**Figure 1:**
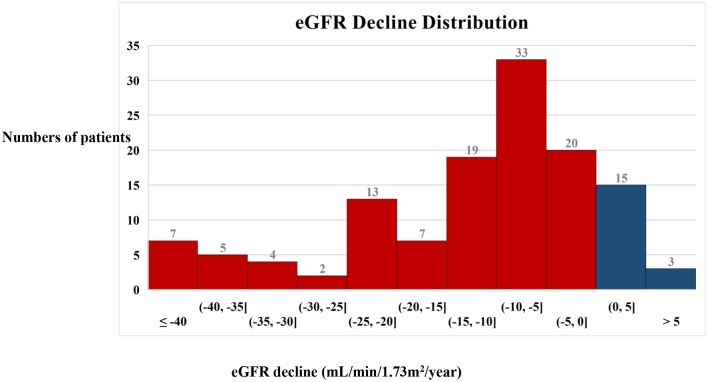
Distribution of the annual decline in eGFR in the patients. A histogram showing the distribution of the annual decline in eGFR in the patients. Blue bars indicate an increase in eGFR and red bars indicate a decrease in eGFR.

A total of 128 eligible patients with type 2 diabetes and biopsy-proven DKD were enrolled in the study. The eGFR slope ranged from 0 to 5 mL/min/1.73 m^2^/year in 15 patients and the eGFR rose by more than 5 mL/min/1.73 m^2^/year in three patients. In most patients (56%), the eGFR slope ranged from 0 to −15 mL/min/1.73 m^2^/year. A total of 30% of patients had an eGFR slope < −15 mL/min/1.73 m^2^/year (Figure 1).

### Clinical and Pathological Characteristics of the Study Cohort

A total of 76% (97/128) of the patients were men and 24% (31/128) were women, and they had a mean age of 51.5 ± 10.7 years. The baseline clinical characteristics of the cohort are shown in Table 1. Briefly, 46% (43/93) of the patients had a family history of diabetes and 29% (37/128) of the patients were current smokers. Their mean systolic blood pressure was 145 ± 23 mmHg and mean diastolic blood pressure was 87 ± 14 mmHg. The median (interquartile range) duration of diabetes was 72 (36, 132) months. A total of 4% (57/128) of the patients had diabetic retinopathy and the mean glycosylated hemoglobin (HbA1c) value was 7.4 ± 1.9%. The mean serum creatinine level was 121 ± 49 μmol/L, the median eGFR was 63 (44, 87) mL/min/1.73 m^2^, and the median proteinuria level was 2.8 (1.3, 5.9) g/24 h.

**Table 1:** Baseline clinical and pathological features of patients with CKD stages 1–5.

**Variables**	**Total (*n* = 128)**	**Slow decliners (*n* = 64)**	**Rapid decliners (*n* = 64)**	***P*-value**
Men (*n*, %)	97 (76)	24 (77)	27 (77)	>0.05
Age (years)	51.5 ± 10.68	52.7 ± 10.1	50.3 ± 11.2	>0.05
Family history of diabetes (*n*, %)	43 (46.2)	14 (30.4)	29 (61.7)	0.002
Body mass index (kg/m^2^)	26.00 ± 4.27 (*n* = 73)	25.48 ± 3.58 (*n* = 35)	26.48 ± 4.82 (*n* = 38)	>0.05
Current Smoker (*n*, %)	37 (29)	12 (39)	10 (29)	>0.05
Systolic blood pressure	145 ± 23	146 ± 21	143 ± 25	>0.05
Diastolic blood pressure	87 ± 14	87 ± 14	87 ± 14	>0.05
Diabetic duration (months)	72 (36, 132)	60 (27, 132)	90 (36, 141)	>0.05
Diabetic retinopathy (*n*, %)	57 (45)	11 (36)	11 (32)	>0.05
Fasting blood glucose (mmol/L)	7.9 ± 3.4	8.2 ± 3.7	7.6 ± 3.1	>0.05
Glycosylated hemoglobin (%)	7.4 ± 1.9	7.5 ± 1.9	7.4 ± 1.9	>0.05
Cystatin-C (mg//L)	1.53 ± 0.55	1.44 ± 0.52	1.62 ± 0.56	>0.05
Serum creatinine (μmol/L)	121 ± 49	118 ± 53	123 ± 45	>0.05
eGFR (mL/min/1.73 m^2^)	63 (44, 87)	64 (43, 95)	59 (45, 83)	>0.05
Albumin to creatinine ratio (mg/g)	1477 (446, 3,242) (*n* = 64)	968 (291, 1,790) (*n* = 35)	2175 (999, 4,903) (*n* = 28)	0.01
Proteinuria (g/24 h)	2.8 (1.3, 5.9) (*n* = 113)	1.7 (0.7, 3.5) (*n* = 54)	4.4 (2.4, 6.8) (*n* = 59)	<0.001
Triglyceride (mmol/L)	2.3 ± 1.9	2.5 ± 2.4	2.0 ± 1.3	>0.05
Total cholesterol (mmol/L)	5.0 ± 1.5	4.9 ± 1.5	5.1 ± 1.5	>0.05
LDL-C	2.9 ± 1.1	2.8 ± 1.1	3.0 ± 1.1	>0.05
Uric acid (mmol/L)	392 ± 86	395 ± 89	388 ± 82	>0.05
**Pathological lesions**				
Glomerular class				0.008
I	13	9	4	
IIa	34	20	14	
IIb	15	11	4	
III	49	17	32	
IV	17	7	10	
IFTA				0.033
0	7	6	1	
1	57	31	26	
2	55	24	31	
3	9	3	6	
Interstitial inflammation				<0.001
0	12	11	1	
1	96	49	47	
2	20	4	16	
Arteriolar hyalinosis				0.472
0	23	14	9	
1	66	31	35	
2	39	19	20	
**Treatment**				
RAAS inhibitors (*n*, %)	106 (83)	28 (90)	31 (89)	0.064
Oral antihyperglycemic drugs (*n*, %)	64 (50)	23 (74)	19 (54)	<0.001
Insulin use (*n*, %)	87 (68)	19 (61)	25 (71)	>0.05
Statins	73 (57)	17 (55)	23 (66)	>0.05
**Follow-up data**				
Follow-up duration (years)	2.0 (1.4, 3.2)	2.4 (1.6, 3.7)	1.6 (1.2, 2.3)	0.001
Measurements (times)	4 (3, 7)	5 (3, 8)	4 (3, 6)	0.014
eGFR slope (mL/min/1.73 m^2^/year)	−8.1 (−18.6, −3.0)	−3.1 (−6.6, 0.2)	−18.6(−12.1,−30.2)	<0.001
eGFR (at end of follow-up)	34 (12, 67)	49 (37, 90)	15 (10, 33)	<0.001
eGFR ≤ 15 (*n*, %)	40 (31.3)	8 (12.5)	32 (50.0)	<0.001

*Data are presented as the mean ± standard deviation, the median with range, or counts and percentages. eGFR, estimated glomerular filtration rate; LDL, low density lipoprotein; IFTA, interstitial fibrosis and tubular atrophy; RAAS, renin-angiotensin-aldosterone system. Two-tailed P < 0.05 was considered statistically significant*.

All the patients in the study underwent a kidney biopsy. Their glomerular lesions were classified as follows: 13 patients had glomerular basement membrane thickening only and were classified as class I; 49 patients had mild or severe mesangial expansion, but no nodular sclerosis (Kimmelstiel-Wilson lesion), and were classified as class II; 49 patients who did not meet the criteria for class IV, with at least one convincing Kimmelstiel-Wilson lesion, were classified as class III; and 17 patients with global glomerular sclerosis in ≥50% of their glomeruli were classified as class IV. For the IFTA score, 7, 57, 55, and 9 patients were scored as 0, 1, 2, and 3, respectively. With regard to interstitial inflammation, 12, 96, and 20 patients were scored as 0, 1, and 2, respectively. For arteriolar hyalinosis, 23, 66, and 39 patients were scored as 0, 1, and 2, respectively.

For the medication administered, 83% (106/128) of the patients received RAAS inhibitor therapy, 50% (64/128) had taken oral antihyperglycemic drugs, 68% (87/128) had injected insulin, and 73% (57/128) had taken statins.

The median eGFR slope was −3.1 (−6.6, 0.2) mL/min/1.73 m^2^/year in slow decliners and −18.6 (−12.2, −30.2) mL/min/1.73 m^2^/year in rapid decliners. Baseline sex distribution, age, diabetic duration, eGFR, and levels of serum glucose and lipids were comparable in rapid and slow decliners. However, significantly more rapid decliners had a family history of diabetes than did slow decliners (61.7 vs. 30.4%, *P* = 0.002). Rapid decliners had significantly higher degree of proteinuria [4.4 (2.4, 6.8) vs. 1.7 (0.7, 3.5), *P* < 0.001], advanced glomerular class (*P* = 0.008) and higher scores of IFTA (*P* = 0.033) and interstitial inflammation (*P* < 0.001) than did slow decliners. Significantly fewer rapid decliners were taking oral antihyperglycemic drugs than slow decliners (54 vs. 74%, *P* < 0.001). However, the use of insulin and statins was similar in the two groups.

### Follow-Up Characteristics

**Figure 2:**
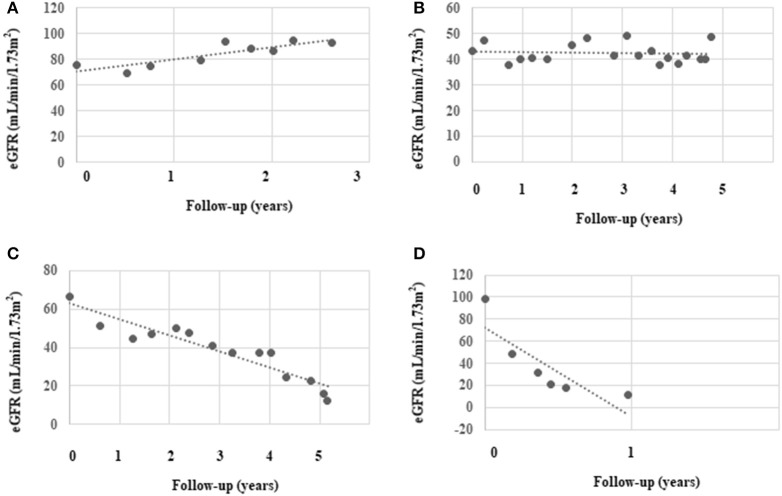
Classical eGFR decline patterns identified in the study. We chose four representative patients from the study cohort to depict their patterns of eGFR decline: **(A)** slow increase in eGFR, **(B)** stable eGFR, **(C)** slow decline in eGFR, and **(D)** rapid decline in eGFR. The black dots represent individual measurements and the dashed lines summarize the trend for each patient.

During a median follow-up period of 2 (1.4, 3.2) years, the median number of serum creatinine measurements made was four (3, 7) times, and the median eGFR slope was −8.1(−18.6, −3.0) mL/min/1.73 m^2^/year. At the end of the follow-up, significantly more rapid decliners progressed into ESKD (50 vs. 13%, *P* < 0.001). Four classical eGFR patterns were identified in our cohort as follows: (a) the eGFR trajectory gradually rose; (b) the eGFR trajectory remained stable; (c) the eGFR trajectory gradually declined; (d) the eGFR trajectory rapidly declined (Figure 2).

### eGFR Decline in the Various Subgroups

**Figure 3:**
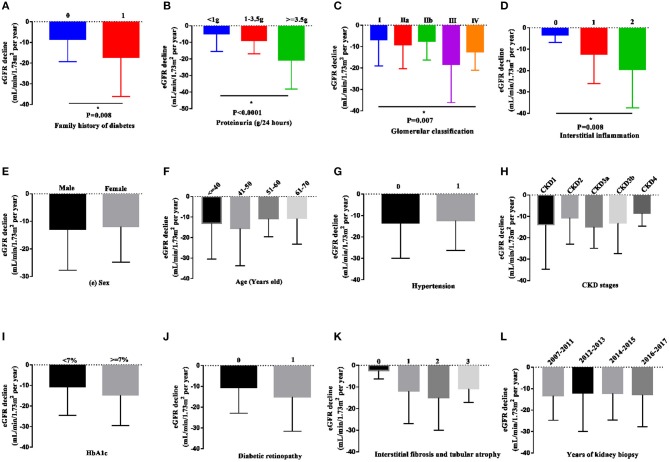
eGFR decline in patients in the various subgroups. Data are presented as the median and interquartile range. The decline in eGFR was significantly more rapid in patients with a family history of diabetes in first-degree relatives, heavier proteinuria, higher grades of glomerular pathology class, and interstitial inflammation **(A–D)**. However, the decline in eGFR was not observed in other subgroups **(E–L)**. Two-tailed *P* < 0.05 was considered statistically significant.

We further compared the decline in eGFR in the various subgroups (Figure 3 and Supplementary Table 1). The eGFR slope was significantly steeper in patients with a family history of diabetes (−17.12 ± 19.01 vs. −8.57 ± 10.65 mL/min/1.73 m^2^/year, *P* < 0.05), higher degree of proteinuria (1, 1–3.5 vs. 3.5g: −4.89 ± 10.57, −8.84 ± 7.93 vs. −20.65 ± 17.54 mL/min/1.73 m^2^/year, respectively, *P* < 0.001), higher grades of glomerular pathology class (I, IIa, IIb, III, and IV: −6.75 ± 12.29, −9.05 ± 11.23, −18.19 ± 17.93, and −12.42 ± 8.66 mL/min/1.73 m^2^/year, respectively, *P* < 0.05) and a higher interstitial inflammation score (0, 1 vs. 2: −3.26 ± 3.59, −12.30 ± 13.85 vs. −19.39 ± 18.02 mL/min/1.73 m^2^/year, *P* < 0.05). There were no significant differences in sex, age, the presence of hypertension, baseline CKD stages, HbA1c, diabetic retinopathy, or IFTA among the subgroups.

### Risk Factors Associated With a Rapid eGFR Decline

We next investigated the association between potential risk factors and a decline in eGFR. Univariable logistic regression showed that a family history of diabetes [odds ratio (OR) 3.683, 95% confidence interval (CI) 1.558–8.706, *P* = 0.003], higher degree of proteinuria (OR 1.263, 95%CI 1.097–1.454, *P* = 0.001), higher grades of glomerular pathology class (OR 1.470, 95%CI 1.097–1.969, *P* = 0.010), higher IFTA (OR 1.793, 95%CI 1.068–3.010, *P* = 0.027), and more interstitial inflammation (OR 5.552, 95%CI 2.122–14.528, *P* < 0.001) were associated with a rapid decline in eGFR. Furthermore, multivariable logistic regression indicated that a family history of diabetes (OR 3.973, 95% CI 1.220–12.931, *P* = 0.022) and higher degree of proteinuria (OR 1.159, 95%CI 1.006–1.336, *P* = 0.041) were independently associated with a rapid decline in eGFR. However, glomerular pathology class, IFTA, and interstitial inflammation were not independently associated with a rapid decline in eGFR (Table 2).

**Table 2:** Logistic regression analyses of patients who showed a rapid or slow decline in eGFR.

**Variables**	**Univariable**			**Multivariable**		
	**Odds ratio**	**95% CI**	***P***	**Odds ratio**	**95% CI**	***P***
Man	1.292	0.574–2.909	>0.05	1.033	0.313–3.405	>0.05
Age	0.979	0.947–1.012	>0.05	1.016	0.959–1.077	>0.05
Family history of diabetes	3.683	1.558–8.706	0.003	3.973	1.220–12.931	0.022
Proteinuria	1.263	1.097–1.454	0.001	1.159	1.006–1.336	0.041
eGFR	0.995	0.983–1.008	>0.05	1.018	0.991–1.045	>0.05
Glomerular class	1.470	1.097–1.969	0.010	1.267	0.771–2.081	>0.05
IFTA	1.793	1.068–3.010	0.027	1.547	0.542–4.414	>0.05
Interstitial inflammation	5.552	2.122–14.528	<0.001	5.394	0.991–29.341	>0.05

*eGFR, estimated glomerular filtration rate; IFTA, interstitial fibrosis and tubular atrophy; CI, confidence interval. Two-tailed P < 0.05 was considered statistically significant*.

## Discussion

In the present study, the median eGFR slope was −8.1 mL/min/1.73 m^2^/year. A total of 70% of the patients showed a decline in the eGFR of more than 5 mL/min/1.73 m^2^/year. Patients with a family history of diabetes, nephrotic-range proteinuria, higher glomerular pathology class, and interstitial inflammation showed a more rapid decline in eGFR. Furthermore, a family history of diabetes was independently associated with a rapid decline in eGFR in our relatively young population.

DKD is a heterogeneous disease that has many clinicopathological manifestations, which are associated with various risk factors. These risk factors can be classified as those relating to susceptibility, initiation, and progression. The following factors are considered to influence progression: ethnicity; systemic conditions, including control of hyperglycemia, overweight, and hypertension; factors leading to acute kidney injury (AKI); and high protein intake (15). However, these risk factors cannot completely explain the rapid progression of DKD in the current study cohort. Therefore, we aimed to identify any additional risk factors for progression of DKD to facilitate timely and effective therapeutic interventions.

Familial clustering of DKD is a well-established susceptibility factor of developing DKD (16, 17). A recent study showed that a rapid decline in eGFR in first-degree relatives increased the risk of a rapid decline in eGFR in patients with diabetes (9). However, whether a family history of diabetes is a risk factor for the progression of DKD remains to be determined. In the current study, we expanded on the findings of previous studies by showing that a family history of diabetes is an independent risk factor associated with a rapid decline in eGFR. This finding of a contribution of a family history of diabetes suggests that the genetic background of diabetes, as well as life style, contribute to a rapid decline in eGFR in patients with type 2 diabetes and DKD.

Genome-wide association and whole-exome sequencing studies have identified a large number of loci that are associated with a higher risk of diabetes and kidney dysfunction (18). A recent study of 6,330 Chinese patients with type 2 diabetes identified five genetic loci (rs11803049, rs911119, rs1933182, rs11123170, and rs889472) that could be used to improve prediction of the rate of eGFR decline (10). Our previous study also showed that variants of the *COL4A3* gene were associated with worse kidney function and heavier proteinuria in patients with maturity onset diabetes of the young (11). Moreover, a recent study that was conducted in 20,791 patients with type 2 diabetes and 24,440 controls showed dozens of susceptibility genes and single variants that were associated with type 2 diabetes (19). Among these genes, some of them are also expressed in the kidney, including the three genes *MC4R, PAM*, and *SLC30A8*, which have the highest significant levels. Although the underlying mechanisms are unclear, we speculate that these risk loci of diabetes might also be associated with the risk of kidney disease. However, in addition to having the same genetic background, families share a similar life style and dietary style. Therefore, clinicians should pay close attention to patients with DKD and a family history of diabetes, because a decline in their eGFR might be rapid.

Nephrotic-range proteinuria is a well-established risk factor for poor renal outcome in DKD and remains an essential tool for monitoring progression and risk stratification of DKD. Progression to massive proteinuria is considered to be the “point of no return.” The Second Joslin Kidney study (8) (type 2 diabetes) reported that 7% of patients with normal albuminuria were rapid eGFR decliners (eGFR decline >5 mL/min/1.73 m^2^/year), 15% of patients with microalbuminuria were rapid decliners and 51% of patients with macroproteinuria were rapid decliners. Similarly, the UKPDS 64 published a study in 2003, which also enrolled relatively younger patients with type 2 diabetes as current study, showed that macroalbuminuria was a strong risk factor for cardiovascular disease and ESKD (20). In the current cohort, the mean decline in eGFR was 4.89 mL/min/1.73 m^2^/year in patients with mild proteinuria, and 20.65 mL/min/1.73 m^2^/year in patients with nephrotic-range proteinuria. The mean decline in eGFR in patients with nephrotic-range proteinuria was twice that of patients with moderate proteinuria. The high incidence of nephrotic-range proteinuria could partially explain the steep decline in eGFR identified in the present cohort. However, patients in the current study were relatively young. Currently, patients with diabetics are becoming progressively older and elderly in many countries. Additionally, these patients are frequently with normal or non-albuminuria in their DKD progression, even in the progression to ESKD by the end (21). Therefore, caution should be used in extending these findings to the diabetic population as whole.

No available animal model closely mimics the progressive renal decline of DKD that occurs in humans. Therefore, the associations between a decline in eGFR and glomerular lesions, IFTA, interstitial inflammation, and arteriolar hyalinosis are poorly defined (8). To the best of our knowledge, this is the first study to determine the characteristics of pathological lesions present in rapid eGFR decliners. Glomerular pathology class, IFTA, and interstitial inflammation were associated with the rate of eGFR decline in the current cohort. A study conducted in Hong Kong of 37 Chinese patients with biopsy-proven DKD also showed that most patients with glomerulosclerosis had a rapid decline in eGFR (10). The underlying mechanisms of glomerulosclerosis are complex and include ischemia of the glomeruli, global deflation, and arteriosclerosis induced by disequilibrium in glomerular hemodynamics, and low-level inflammation (8). With gradually increasing awareness of normoalbuminuric DKD and AKI in patients with diabetes, the conventional glomerulus-centered concept of renal pathophysiology has been expanded to include tubular-interstitial injury (22, 23). Multiple studies have shown that diabetes is an independent risk factor for AKI (24, 25) and the kidneys are likely to become vulnerable to a “second hit” in a high-glucose environment. After acute injury, failure to fully compensate for functional impairment might cause a more rapidly progression to ESKD (26). Therefore, AKI may accelerate a decline in eGFR, but this may be imperceptibles for some time.

Four patterns of classical trajectories of eGFR decline were identified in the present study (Figure 2). Interestingly, in two patients, the eGFR actually gradually rose by ~10 mL/min/1.73 m^2^/year. The first of these two patients is a 40-year-old man whose eGFR increased from 110 mL/min/1.73 m^2^ and to 133 mL/min/1.73 m^2^ and then decreased to 120 mL/min/1.73 m^2^ over 2.3 years. We believe that he experienced hyperfiltration. The second patient was a 63-year-old woman who had an eGFR that was stable at ~70 mL/min/1.73 m^2^ for 1 year, and it then increased to ~90 mL/min/1.73 m^2^ (Figure 2A), where it remained at the end of the study. She was taking RAAS inhibitors throughout the study period and we speculate that she was sensitive to treatment, which induced improvement of hemodynamics. The fastest decline in eGFR in the current study was 81.3 mL/min/1.73 m^2^/year (baseline eGFR was ~90 mL/min/1.73 m^2^/year) in a patient (47-year-old man) who was undergoing hemodialysis until now (Figure 2D). The fastest eGFR decline in the Joslin Kidney study was 72 mL/min/1.73 m^2^/year. Notably, the glomerular pathology class of the first two patients (whose eGFR rose) was IIa and I, respectively. However, the fastest decliner's glomerular pathology class was III, and he also had a higher tubular-interstitial injury score. These findings suggest that kidney function may be restored by intensive treatment in patients with a mild pathology.

There are some limitations of the study that should be discussed. First, there was likely to be selection bias and low number of older or elderly patients were enrolled. This was a retrospective study and the sample size was relatively small. However, we tried to minimize potential confounding by the inclusion of patients with kidney diseases other than DKD. Additionally, to minimize the effect of methodological differences between laboratories, creatinine data from other hospitals were excluded. Second, the follow-up period was relatively short and we nephrologists had difficulty accessing data from patients with diabetes, but no kidney phenotype. However, we calculated the eGFR changes in eGFR in patients who had advanced DKD and identified the risk factors for its progression. Third, our cohort only included only patients from Sichuan Province. There were also other variables that were not assessed in the present study, including medical care maturity and educational level, which might affect the prognosis of DKD. Therefore, larger-scale studies of a broader population should be performed to confirm our findings.

## Conclusion

A family history of diabetes in first-degree relatives is independently associated with a rapid decline in eGFR in the current relatively young patients. These findings suggested that early diagnosis and treatment should be performed in these patients.

## Data Availability Statement

All datasets generated for this study are included in the article/Supplementary Material.

## Ethics Statement

The studies involving human participants were reviewed and approved by the Ethics Committee of West China Hospital. The patients/participants provided their written informed consent to participate in this study.

## Author Contributions

YWa and FL organized the project. YWa and LZ analyzed data and wrote the manuscript. All other authors took part in collecting data, as well as double checking data and the manuscript.

### Conflict of Interest

The authors declare that the research was conducted in the absence of any commercial or financial relationships that could be construed as a potential conflict of interest.
