# Supplementary material for: Implications of a Family History of Diabetes and Rapid eGFR Decline in Patients With Type 2 Diabetes and Biopsy-Proven Diabetic Kidney Disease
Source: Front Endocrinol (Lausanne). 2019 Dec 13;10:855. doi: 10.3389/fendo.2019.00855 (PMC6923196; doi:10.3389/fendo.2019.00855)
Supplement: Supplementary file 1 [file Table_1.docx]

| Family history of diabetes | No  n=50 | Yes  n=43 | *P* value |
| --- | --- | --- | --- |
| eGFR decline (mL/min/1.73m^2^  per year) | -8.565±10.65 | -17.12±19.01 | <0.05 |

Supplementary Table1. eGFR decline in subgroups

| Proteinuria | <1 g  n=22 | 1-3.5g  n=41 | >=3.5g  n=50 | *P* value |
| --- | --- | --- | --- | --- |
| eGFR decline (mL/min/1.73m^2^  per year) | -4.89±10.57 | -8.84±7.93 | -20.65±17.54 | <0.05 |

| Glomerular classification | I  n=13 | IIa  n=34 | IIb  n=15 | III  n=49 | IV  n=17 | *P* value |
| --- | --- | --- | --- | --- | --- | --- |
| eGFR decline (mL/min/1.73m^2^  per year) | -6.75±12.29 | -9.05±11.23 | -7.35±8.87 | -18.19±17.93 | -12.42±8.66 | <0.05 |

| Interstitial inflammation | 0  n=12 | 1  n=96 | 2  n=20 | *P* value |
| --- | --- | --- | --- | --- |
| eGFR decline (mL/min/1.73m^2^  per year) | -3.26±3.59 | -12.30±13.85 | -19.39±18.02 | <0.05 |

| Sex | Male  n=97 | Female  n=31 | *P* value |
| --- | --- | --- | --- |
| eGFR decline (mL/min/1.73m^2^  per year) | -12.79±14.96 | -11.86±12.9 | >0.05 |

| Age | <=40  n=18 | 41-50  n=41 | 51-60  n=39 | 61-70  n=30 | *P* value |
| --- | --- | --- | --- | --- | --- |
| eGFR decline (mL/min/1.73m^2^  per year) | -12.99±17.49 | -15.49±18.19 | -10.77±8.765 | -10.63±12.54 | >0.05 |

| Hypertension | 0  n=26 | 1  n=102 | *P* value |
| --- | --- | --- | --- |
| eGFR decline (mL/min/1.73m^2^  per year) | -13.38±16.5 | -12.35±13.96 | >0.05 |

| CKD stages | 1  n=31 | 2  n=35 | 3a  n=28 | 3b  n=23 | 4  n=11 | *P* value |
| --- | --- | --- | --- | --- | --- | --- |
| eGFR decline (mL/min/1.73m^2^  per year) | -13.8±20.9 | -10.6±12.4 | -14.9±10.0 | -13.1±14.3 | -8.5±6.1 | <0.05 |

| HbA1c | <7%  n=53 | >7%  n=48 | *P* value |
| --- | --- | --- | --- |
| eGFR decline (mL/min/1.73m^2^  per year) | -10.5±14 | -14.64±14.78 | >0.05 |

| Diabetic retinopathy | 0  n=69 | 1  n=57 | *P* value |
| --- | --- | --- | --- |
| eGFR decline (mL/min/1.73m^2^  per year) | -10.49±12.4 | -15.02±16.57 | >0.05 |

| Interstitial fibrosis and tubular atrophy | 0  n=7 | 1  n=57 | 2  n=55 | 3  n=9 | *P* value |
| --- | --- | --- | --- | --- | --- |
| eGFR decline (mL/min/1.73m^2^  per year) | -2.326±3.976 | -11.85±15.04 | -14.89±15.12 | -10.79±6.358 | >0.05 |

| Years | 2007-2011  n=17 | 2012-2013  n=23 | 2014-2015  n=31 | 2016-2017  n=57 | *P* value |
| --- | --- | --- | --- | --- | --- |
| eGFR decline (mL/min/1.73m^2^  per year) | -13.22±11.49 | -12.09±17.84 | -12.12±12.54 | -12.8±15.03 | >0.05 |
